# Exploring Sarcopenic Obesity in the Cancer Setting: Insights from the National Health and Nutrition Examination Survey on Prognosis and Predictors Using Machine Learning

**DOI:** 10.3390/bioengineering12090921

**Published:** 2025-08-27

**Authors:** Yinuo Jiang, Wenjie Jiang, Qun Wang, Ting Wei, Lawrence Wing Chi Chan

**Affiliations:** 1 Department of Health Technology and Informatics, The Hong Kong Polytechnic University, Hong Kong SAR, China; 2 Department of Oncology, Zhujiang Hospital, Southern Medical University, Guangzhou 510280, China; 3 Department of Critical Care Medicine, Yueyang People’s Hospital, Yueyang 414000, China; 4 Department of Emergency, Zhujiang Hospital, Southern Medical University, Guangzhou 510280, China

**Keywords:** sarcopenic obesity, cancer, prognosis, machine learning

## Abstract

**Objective**: Sarcopenic obesity (SO) is a combination of depleted skeletal muscle mass and obesity, with a high prevalence, undetected onset, challenging diagnosis, and poor prognosis. However, studies on SO in cancer settings are limited. We aimed to explore the association between SO and mortality and to investigate potential predictors involved in the development of SO, with a further objective of constructing a model to detect its occurrence in cancer patients. **Methods**: The data of 1432 cancer patients from the National Health and Nutrition Examination Survey (NHANES) from the years 1999 to 2006 and 2011 to 2016 were included. For survival analysis, univariable and multivariable Cox proportional hazard models were used to examine the associations of SO with overall survival, adjusting for potential confounders. For machine learning, six algorithms, including logistic regression, stepwise logistic regression, least absolute shrinkage and selection operator (LASSO), support vector machine (SVM), random forest (RF), and extreme gradient boosting (XGBoost), were utilized to build models to predict the presence of SO. The predictive performances of each model were evaluated. **Results**: From six machine learning algorithms, cancer patients with SO were significantly associated with a higher risk of all-cause mortality (adjusted HR 1.368, 95%CI 1.107–1.690) compared with individuals without SO. Among the six machine learning algorithms, the optimal LASSO model achieved the highest area under the curve (AUC) of 0.891 on the training set and 0.873 on the test set, outperforming the other five machine learning algorithms. **Conclusions**: SO is a significant risk factor for the prognosis of cancer patients. Our constructed LASSO model to predict the presence of SO is an effective tool for clinical practice. This study is the first to utilize machine learning to explore the predictors of SO among cancer populations, providing valuable insights for future research.

## 1. Introduction

Sarcopenia is characterized by a significant depletion of skeletal muscle mass and strength, accompanied by impairment in physical function. The coexistence of sarcopenia and obesity is termed sarcopenic obesity (SO) [1,2]. When the muscle mass is lost while fat is gained simultaneously, even though the weight change is inconspicuous, body composition is altered: the accumulation of body fat may cover up the loss of muscle mass. The chronic progression of sarcopenia and obesity makes symptoms more insidious [3,4]. Additionally, due to the synergistic and superimposed effects of sarcopenia and obesity [5], SO’s adverse health-related consequences may be greater than the sum of respective poor outcomes of sarcopenia or obesity alone [2,6,7].

Although a recent study by Lian et al. developed a machine learning model to predict SO in aging communities and nursing populations, it did not construct a predictive model especially for cancer individuals [8]. Existing research shows that cancer patients with SO are at special risk for adverse outcomes, leading to a reduction in quality of life, complications after surgical resection, increased risk of dose-limiting toxicity during chemotherapy, and increased mortality [9]. However, among the few available studies on SO among cancer populations, most have limited sample sizes or merely concentrate on prognosis or surgical complications in patients with SO in each single cancer setting [10]. There are fewer large and national sample studies on SO in pan-cancer settings. Our study could fill these research gaps.

Although proposed in previous systematic reviews, potential predictors of SO still remain hypothetical. It must be noted that up to now, no epidemiological or clinical evidence has been observationally reported about which factors are associated with the incidence of SO in cancer patients. Therefore, this present study is the first study aimed to explore the clinical implications of SO and the possible predictors associated with SO in patients with malignant tumors by utilizing large and representative pan-cancer samples with long follow-up time from The National Health and Nutrition Examination Survey (NHANES).

## 2. Materials and Methods

### 2.1. Data Source and Sample Collection

NHANES is a national and representative survey of studies designed to assess the health and nutritional status of residents in the United States. Participants were selected by using a complex multi-stage probability sampling design.

Here is what makes this survey unique: it combines interviews and medical examinations. Sociodemographic, dietary data, and other basic information were collected during the interview first; medical, dental, and physiological measurements, as well as laboratory testing, were subsequently administered in the mobile examination center (MEC). All data were recorded and coded in the NHANES database. This survey then became a continuous program to satisfy the growing demands. The NHANES program was approved by the National Center for Health Statistics research ethics review board, and informed consent was obtained from all included participants.

Mortality data were ascertained by probabilistic matches between the NHANES database and the US National Death Index, with causes of death recorded by the International Classification of Diseases. The overall survival (OS) time was calculated as the difference between the age at the in-home interview and the time of cancer diagnosis, plus the duration of follow-up. More details are documented at https://www.cdc.gov/nchs/nhanes/, accessed on 26 August 2025).

**Figure 1:**
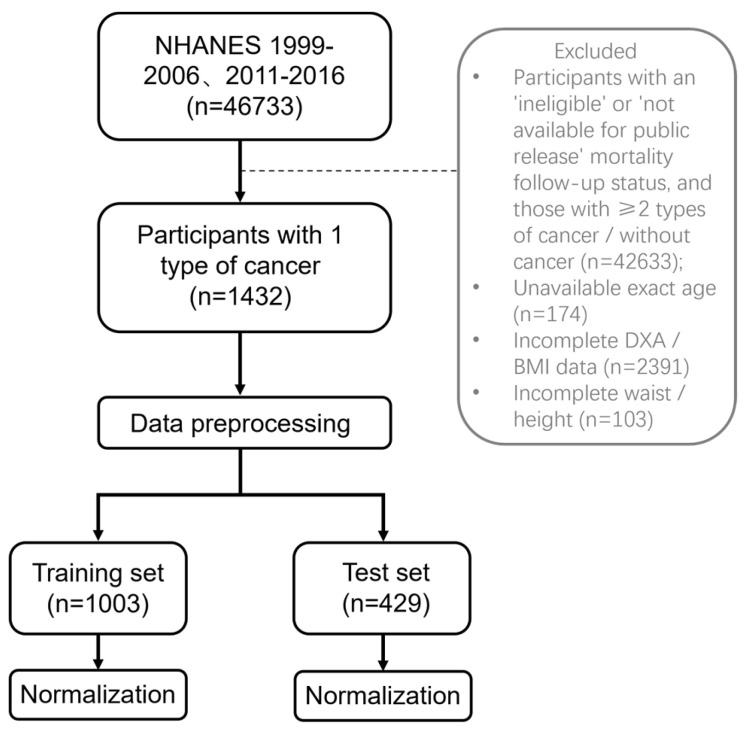
Flow chart.

For this cross-sectional study, we merged available data from 1999 to 2006 and 2011 to 2016. All included participants underwent dual-energy X-ray absorptiometry (DXA) measurements. Exclusion criteria were (1) status for mortality follow-up ineligible and not available for public release, (2) participants with ≥2 types of cancer or without cancer, (3) participants with age recorded as “80 years or older” or “85 years or older”, resulting in unavailable exact age, (4) incomplete DXA or BMI data, (5) incomplete waist circumference or height data. After the screening, there were 1432 participants included (Figure 1). The reason for excluding 2007 to 2010 is the absence of whole-body DXA data; only femur and spine data are available. The cancer types includes bladder, blood, bone, brain, breast, cervix, colon, esophagus, gallbladder, kidney, larynx/ trachea, leukemia, liver, lung, lymphoma/Hodgkin’s disease, melanoma, mouth/tongue/lip, nervous system, ovary, pancreas, prostate, rectum, skin (non-melanoma), skin (unknown), soft tissue (muscle or fat), stomach, testis, thyroid, uterus, other.

### 2.2. DXA and the Definition of SO

DXA has the advantage of accuracy and no contraindications except for pregnancy [11,12,13]. It should be performed no more than twice per year. Until now, DXA has become one of the widely accepted and applied body composition measurements in clinical work. We used the released DXA data recorded by certified technologists from the NHANES website.

According to the Foundation for the National Institutes of Health (FNIH) criteria, sarcopenia is defined as the sum of lean mass for all four extremities (arms and legs) adjusted for BMI (<0.789 kg for males, <0.512 kg for females) [14,15].

We used relative fat mass (RFM), a new estimator based on sex, height, and waist circumference, to assess obesity and body fat percentage, which has been verified as superior to BMI [16,17]. The formula was as follows: 64 − (20 × height/waist circumference) + (12 × sex), sex = 0 for men and 1 for women. RFM cutoffs are 40% for women and 30% for men to diagnose obesity [18]. The use of the conventional BMI index to define obesity is not perfect. BMI is based solely on height and weight measurements, without taking into account the variations in body fat content, distribution, and muscle mass composition. Unlike BMI, RFM is a straightforward linear equation based on height, waist circumference, and sex. Its simplicity allows for easy data collection and calculation without the necessity for complex computations or specialized equipment. Notably, the formulation of RFM is on the basis of DXA, which is consistent with the measurement method employed in our research, enhancing its reliability and comparability. Moreover, RFM demonstrates a lower misclassification rate for obesity compared to BMI, thereby improving the accuracy of classification among the population.

Participants with cancer who have the coexistence of sarcopenia and obesity were defined as SO. For one thing, we could divide the whole cancer cohort into two categories: SO and Non-SO. For another thing, four groups were classified depending on the combination of sarcopenia and obesity:

1. Non-sarcopenia with non-obesity (nS-nO);
2. Non-sarcopenia with obesity (nS-O);
3. Sarcopenia with non-obesity (S-nO);
4. Sarcopenia with obesity (S-O).

### 2.3. Statistical Analysis

Sample weights were not analyzed in this study because NHANES sample weights were generated to reflect the distribution in the general US population, which differs from the target population of this study. We concentrated on participants with only one type of cancer.

Cox proportional hazard models were used to evaluate the association between SO and mortality rate. Hazard ratios (HRs) and corresponding 95% confidence intervals (CIs) were calculated in univariable and multivariable Cox models to estimate the risk of all-cause mortality in cancer individuals with SO. Multivariable models were constructed in three stages by adjusting for different covariates selected from univariate models. Model 1 was the crude model and was unadjusted, model 2 was additionally adjusted for sex and age, and model 3 was adjusted for variables in model 2 plus other sociodemographic characteristics (including race, education, and marital status). The Kaplan–Meier curve was used to show the survival probability, and the Log-rank method was used to compare the survival curves of multiple groups. Additionally, we further conducted the stratified analysis by categorizing cancer types according to systems. The survival analysis methods for patients with different system-specific tumors were consistent with those used previously.

In the year cycle 1999–2006, a significant amount of DXA data was missing, along with systematic and non-random patterns. To minimize potential bias and provide a complete dataset, these missing values were imputed using multiple-imputation methodology by NHANES technical staff. After imputation, five sets of measured and imputed values are available on the official website for download and analysis. Our analysis was based on the five datasets, and the average was calculated across them.

All analyses were performed in R software. The nhanesR package was used to extract, download, and process NHANES data. The survival and survminer packages were employed for survival analysis and visualization. We used stats, glmnet, randomForest, e1071, and xgboost packages for machine learning in R (4.4.1).

Statistical tests were considered significant at *p* < 0.05 (2 tails).

### 2.4. Building Process and Statistical Analysis of the Machine-Learning Models for Predicting SO

#### 2.4.1. Data Preprocessing

In the machine learning algorithmic process, the target variable was the presence of SO in cancer patients, which is a binary categorical variable. More than 450 independent variables were extracted from the NHANES database, including demographics data, examination data (including body measures: weight, height, skinfold thicknesses, circumferences of head, waist, limb, etc.; DXA; physician examinations such as blood pressure), laboratory data (such as blood and urine test results), and questionnaire data (including smoking status and alcohol consumption status data). Due to the raw data being uploaded every biennial cycle, sometimes the same variable differed in the case of the character or had a diverse expression approach and needed to be manually renamed. Additionally, biomarkers with unit duplication, excessively high missing count, or low incidence were excluded. Ultimately, a total of 108 independent variables were selected for final input.

For categorical variables, One-Hot Encoding was used to convert them into an acceptable format for training and testing machine learning models. Z-score standardization was performed to eliminate the scale differences between different features.

#### 2.4.2. Training and Test Sets

A total of 1432 patients with one type of cancer were randomly divided into training and test sets by using a 7:3 split ratio; 1003 patients were allocated to the training set (including 150 SO participants). The remaining 429 participants were allocated to the test set.

#### 2.4.3. Model Building Process

In our study, six commonly used single-model machine learning algorithms—logistic regression, stepwise logistic regression, least absolute shrinkage and selection operator (LASSO), support vector machine (SVM), random forest (RF), and extreme gradient boosting (XGBoost)—were employed to predict the presence of SO. We first used the training set to construct models. After constructing the model using 108 independent variables from the training set, we ranked each feature’s contribution to predicting the presence of SO. Based on the importance ranking, the feature selection process involved choosing the top 10, 15, and 20 features to be reintroduced into the respective models. We retrained the model using the top 10, 15, and 20 features as inputs and conducted hyperparameter tuning. For the LASSO model, we used 10-fold cross-validation to identify the optimal regularization parameter. For SVM, we evaluated four different kernel functions and ultimately chose the linear kernel, as it showed the best performance. Then, 10-fold cross-validation was employed to evaluate the performance of each C parameter (C parameter =10). For the RF model, we optimized the mtry and ntree parameters by using 10-fold cross-validation to enhance accuracy and reliability (mtry = 130, ntree = 15). Regarding the XGBoost model, a range of parameters, including the number of boosting rounds, maximum depth of trees, learning rate, gamma, sample and feature subsampling proportions, and minimum child weight, were optimized by 10-fold cross-validation (number of boosting rounds = 20, maximum depth of trees = 5, learning rate = 0.1, gamma = 0, colsample_bytree = 1, subsample = 0.7, minimum child weight = 1). We applied the models on the test set to evaluate their predictive performance, assessing the performance on unseen data to evaluate their generalization capability.

#### 2.4.4. Performance Metrics

We employed the receiver operating characteristic (ROC) curve analysis and generated feature importance diagrams and the confusion matrix. Additionally, we calculated the area under the curve (AUC) and 95% confidence intervals for accuracy, sensitivity, and specificity for six machine-learning models.

## 3. Results

### 3.1. Demographics and Characteristics Among Cancer Population

Of the 46,733 adults aged 20–85 of the 7-year cycles from NHANES, a total of 1432 cancer patients with SO (median age, 62 years) were included in this analysis according to the exclusion criteria. Median follow-up was 133 months, during which there were 617 deaths. Of all the cancer patients, 211 participants had SO (14.7%), 621 (43.4%) were male, and the majority were Non-Hispanic White.

In the dichotomous classification, participants in the SO group were older and had higher BMI and RFM values compared to those in the Non-SO group (Table 1).

Under the quadruple taxonomy, individuals in the S-nO group had the highest median age and the highest proportion of males. The nS-O group had the lowest proportion of males and exhibited the highest BMI and RFM values (Table 2).

Under both the dichotomous and quadruple SO classification schemes, Non-Hispanic White individuals represented the largest racial group, and married or cohabiting status was the most common marital status. Regarding educational attainment, the nS-nO group in the quadruple taxonomy and the Non-SO group in the dichotomous classification each exhibited a higher proportion of individuals with education beyond high school. The SO group consistently exhibited a lower poverty-to-income ratio, the lowest proportion of never-smokers, and a higher prevalence of diabetes mellitus under both classification systems (Table 1 and Table 2).

### 3.2. SO and All-Cause Mortality in the Entire Cohort

**Figure 2:**
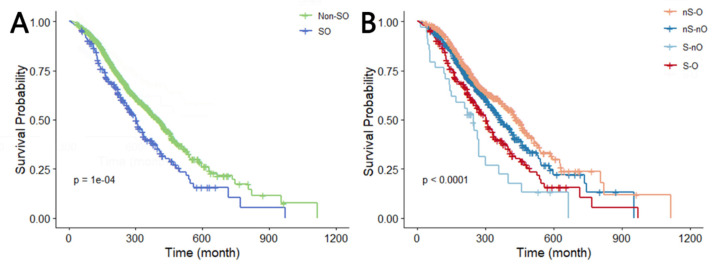
Kaplan–Meier curve of OS (log-rank test: *p* = 0.0001). (**A**,**B**) Survival curves when the total population is divided into two and four categories. Abbreviations: dichotomous taxonomy of SO: SO, sarcopenic obesity; Non-SO, non-sarcopenic obesity; Quadruple taxonomy of SO: nS-nO, non-sarcopenia and non-obesity; nS-O, non-sarcopenia and obesity; S-O, sarcopenia and obesity; S-nO, sarcopenia and non-obesity.

When the whole cancer cohort was divided into two categories, we observed that SO was significantly associated with a higher risk of all-cause mortality in Model 1 (HR 1.476, 95%CI 1.211–1.799) when compared with non-SO patients. After adjustment for age, sex, race, education, and marital status, the association remained similar in Model 3 (HR 1.368, 95%CI 1.107–1.690). Similarly, when the study population was classified into four groups depending on the combination of sarcopenia and obesity, all-cause mortality in the S-O group was 1.364 (95%CI 1.097–1.698) times higher than the nS-nO group in Model 1. This association remained constant after full adjustment in Model 3 (HR 1.298, 95%CI 1.028–1.640) (Table 3). Additionally, Kaplan–Meier curves visually illustrated that the SO group had significantly poorer overall survival compared to the reference group, regardless of how the study population was classified (Figure 2).

We observed that compared with the nS-nO group, HR for all-cause mortality in the nS-O group was consistently less than 1 in models 1–3, which indicated the protective effect of obesity among our participants (Table 3).

### 3.3. SO and All-Cause Mortality in Patients with Different Cancer Systems

The reproductive system group (*n* = 459, including 72 SO cases) consisted of cancer types of the breast, cervix, testis, ovary, and uterus. The integumentary system group (*n* = 432, including 52 SO cases) included melanoma, non-melanoma skin cancers, and skin cancers of unknown type. The digestive system group (*n* = 114, including 19 SO cases) included cancers of the colon, esophagus, gallbladder, liver, stomach, pancreas, rectum, and mouth/tongue/lip. The urinary system group (*n* = 216, including 41 SO cases) included cancers of the bladder, kidney, and prostate. Additionally, there were 73 patients (including 14 SO cases) categorized as having other types of cancer.

Other cancer types were excluded in the stratified analysis due to their limited sample sizes or the insufficient number of diagnosed SO cases within these groups.

The results show that HR and adjusted HR values in models 1, 2, and 3 under the dichotomous classification of SO were generally greater than 1 in reproductive, integumentary, digestive, urinary system group, and other types of cancer, with the exception of the adjusted HR value in model 3 of the urinary system group, which was less than 1 (Table 4, Table 5, Table 6, Table 7 and Table 8). Among these, only the HR and adjusted HR values for model 1 in the reproductive system group (Table 4), models 1 and 2 in the integumentary system group (Table 5), and model 1 in the other types of cancer group (Table 8) were statistically significant. Similarly, under the Quadruple taxonomy of SO, only the HR and adjusted HR values for models 1 and 2 in the reproductive and integumentary system groups (Table 4 and Table 5), and model 1 in the other types of cancer group were statistically significant (Table 8).

### 3.4. Performance of Different Machine Learning Models in Predicting SO

**Figure 3:**
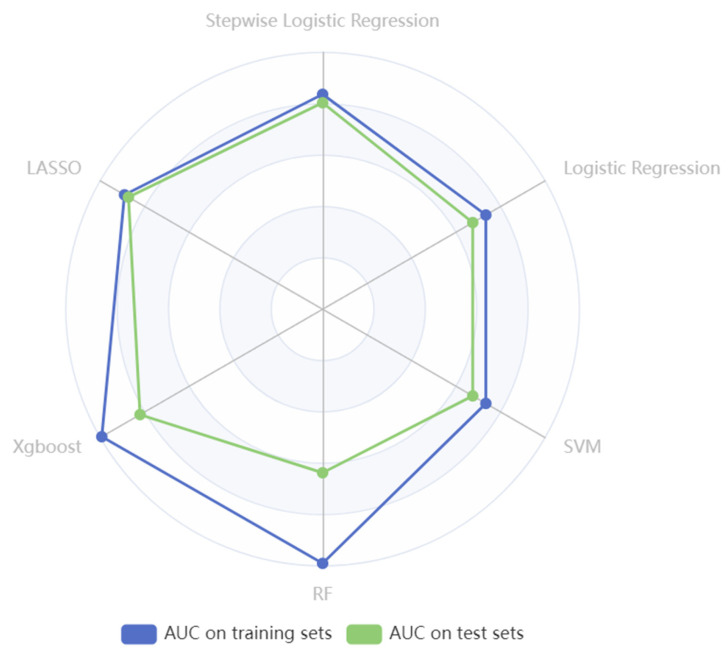
The radar plot illustrates AUC values of six models in identifying SO in cancer settings.

According to the variable selection criteria, we chose 108 variables as the input to train the machine learning models. Predictive performances of SO on the training and test sets are shown in Table 9, and the six models’ optimal AUC values are shown in Figure 3.

Notably, employing the LASSO method for selecting the top 20 features to construct a model resulted in the highest AUC of 0.858 on the test set. Following LASSO, the stepwise logistic regression model (AUC= 0.804 on the test set) and the logistic regression (AUC= 0.675 on the test set) achieved the second and third highest AUC after feature selection. However, the RF model underwent a 10-fold cross-validation process, and the AUC values were 0.990 on the training set and 0.637 on the test set. Similarly, after grid search and 10-fold cross-validation, the AUC values of XGBoost on the training set were 0.993 and 0.821 on the test set. The results of RF and XGBoost indicated overfitting of the training set, as both models demonstrated overly optimistic performance on the training data but showed comparatively poorer performance on the unseen test data (Table 9).

**Figure 4:**
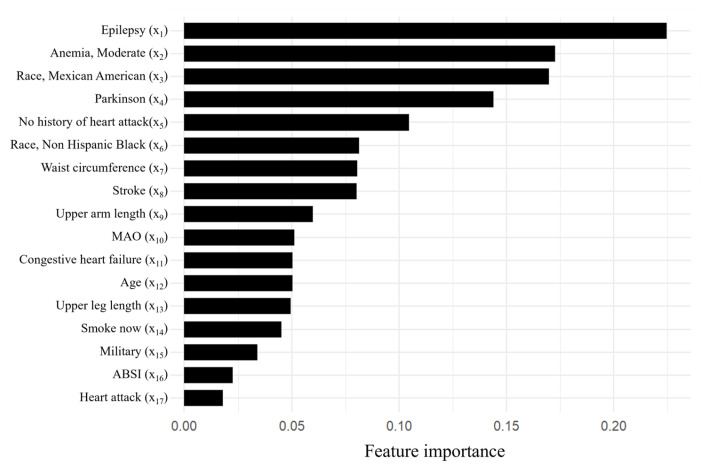
Feature importance of the top 17 variables of the optimal LASSO model.

**Figure 5:**
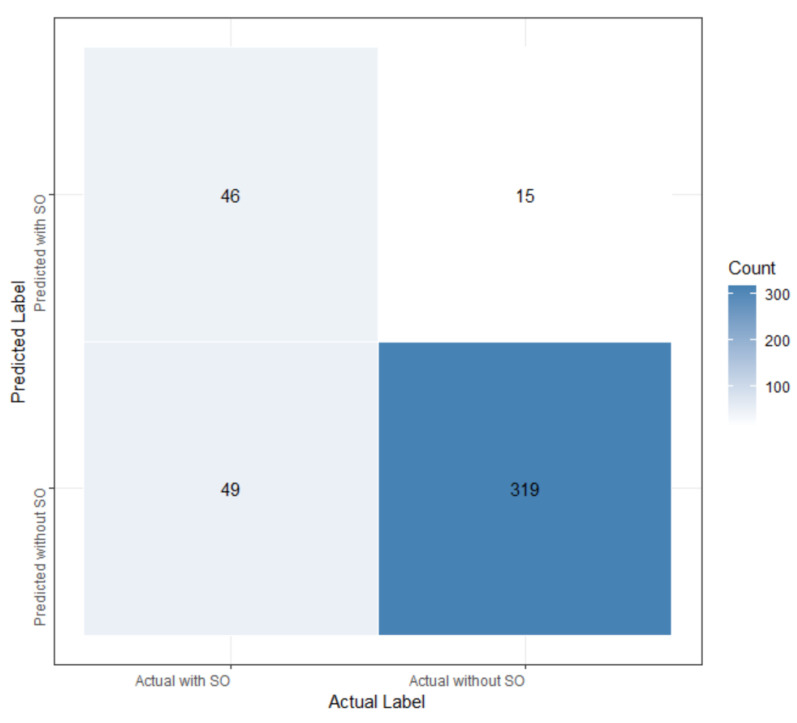
The confusion matrix of the LASSO model.

After the explorations, we focused on the LASSO algorithm. Further investigations were carried out using a different number of features to build LASSO models, employing 10-fold cross-validation to enhance model robustness. Remarkably, LASSO achieved optimal performance with the utilization of the top 17 features, attaining AUC values of 0.891 and 0.873 on the training and test set, respectively, with a sensitivity of 48.4%, a specificity of 95.5%, and an accuracy of 85.1%. Table 10 provides detailed descriptions of the top 17 features in our optimal LASSO model. Figure 4 illustrates the feature importance ranking for predicting SO among cancer participants, while the confusion matrix is presented in Figure 5.

Logit = 0.225 x_1_ + 0.173 x_2_ + 0.17 x_3_ + 0.144 x_4_ − 0.105 x_5_ − 0.081 x_6_ + 0.081 x_7_ − 0.08 x_8_ − 0.06 x_9_ + 0.051 x_10_ − 0.05 x_11_ + 0.05 x_12_ − 0.049 x_13_ − 0.045 x_14_ + 0.034 x_15_ + 0.022 x_16_ + 0.018 x_17_ + 0.304,

where x_i_ represents the normalized value of the ith variable.

**Figure 6:**
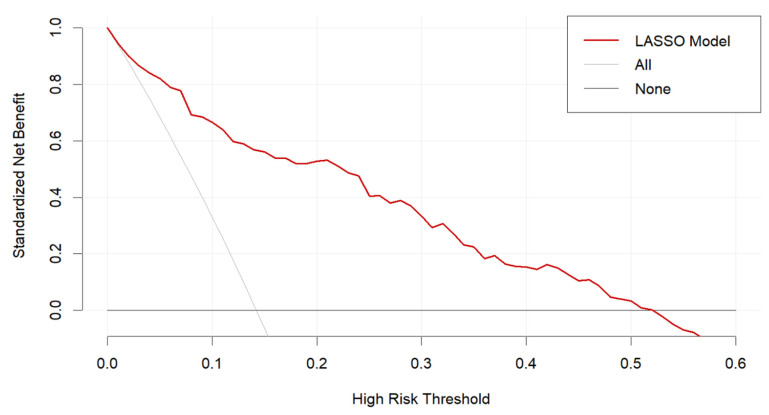
Decision curve analysis for the LASSO model.

From the decision curve analysis (Figure 6), our LASSO model showed a significant net benefit compared with two simple scenarios, all SO or no SO, between a threshold probability range of 0.05 to 0.5. This curve highlights the value of our LASSO model for predicting SO when applied in clinical practice.

## 4. Discussion

### 4.1. Prediction of SO

In this study, we developed an effective model to predict SO among cancer patients by using machine learning. As mentioned before, the SVM model built by Lian R et al. was designed to predict SO in aging communities and nursing populations and achieved an AUC of 0.862 in the internal validation set and 0.785 in the external validation set [8]. In comparison, our model achieved an AUC value of 0.891 and 0.873 in the training and test sets. This suggests that our LASSO model may have a relatively high predictive value in this specific cancer population. The top 17 features selected by the optimal LASSO model can be easily obtained through medical consultations and simple body measurements, without any blood test or invasive operations. Therefore, this predictive tool is convenient for both clinical doctors and patients to utilize, indicating its value in clinical practice.

From our findings, the top 17 features identified by importance ranking in the LASSO model are related to race, existing disease, body measurements data, and lifestyle behavior. However, laboratory data, other medical examination data, and questionnaire data play less important roles in predicting SO. This phenomenon suggests that predictors closely related to the development of SO in cancer patients may primarily involve a combination of genetic and lifestyle influences, providing potential directions for further research on the mechanisms of SO.

### 4.2. The Mechanism of SO in the Development and Progression of Cancer

SO can establish a metabolic and inflammatory environment that facilitates the development and progression of cancer, involving mitochondrial dysfunction, oxidative stress, insulin resistance, hormonal dysregulation, and chronic inflammation. These pathogenic mechanisms are interrelated, like a huge, interconnected network.

Mitochondrial dysfunction in SO results in increased production of reactive oxygen species (ROS), which can trigger oxidative stress [21]. Oxidative stress can accelerate the progression of sarcopenia and lead to DNA damage, thereby increasing the risk of mutations in proto-oncogenes and suppressor genes [22].

SO is associated with insulin resistance (IR), leading to several negative effects. Firstly, IR results in hyperinsulinemia, which promotes cancer development by increasing the uptake of glucose in cancer cells and interacting with the insulin-like growth factor-1 receptor [23]. Secondly, IR inhibits muscle amino acid uptake, while hyperglycemia associated with IR provides a rich source of both amino acids and glucose, thereby supplying the required nutrients to support tumor growth [24,25]. Additionally, IR induces oxidative stress and higher levels of ROS, acting synergistically with mitochondrial dysfunction, further contributing to tumorigenesis [23].

Obesity in SO is associated with elevated levels of estradiol [23], which can induce the proliferation of tumors by enhancing the transcription of oncogenes [26]. Other hormones, such as estrone and testosterone, are stimulators of some tumor cell lines [27]. Additionally, chronic, low-grade, and systemic inflammation mediated by proinflammatory cytokines caused by SO can promote tumor progression [28].

All these mechanisms work together, leading to the acceleration of cancer progression in individuals with SO.

### 4.3. The Influence of Obesity in Cancer Patients with SO

Conclusions derived from previous studies show that excess body weight is an established risk factor and has adverse influences on survival rates and progression in multiple types of cancer [29,30]. Nonetheless, the opposite conclusions were drawn in some findings, showing that being obese is linked to better prognosis in cancer patients, called the ‘obesity paradox’, which conforms with our findings for all-cause mortality in the entire cohort (Table 3) [31].

There are several hypotheses to explain the emergence of the “obesity paradox” [32]. Firstly, adipose tissue can serve as a vital source of energy and nutrients, being metabolized to provide essential nutrition and energy [33]. Non-obese individuals with malignant tumors probably experience insufficient energy reserves against chronic consumption during the advanced stages of the disease, leading to a poorer prognosis. Secondly, obese patients often receive lower drug doses that are relatively lower in proportion to their body weight [34]. While minimizing side effects, it may impact treatment efficacy. Additionally, it is noteworthy that adipocytes can secrete immunomodulatory substances such as leptin and interleukin-10, potentially reducing inflammatory responses and improving survival rates [35,36]. Moreover, the use of BMI as a measure of obesity may not be completely accurate, because some obese patients may disguise symptoms of low muscle mass, and even individuals with the same BMI may have varied body compositions and outcomes [37]. Future research is needed to clarify the pathophysiological mechanisms underlying the obesity paradox.

### 4.4. Novelty and Limitations

Regarding novelty, in the first place, among the available studies on SO in cancer populations, the majority are conducted based on one specific type of cancer, employ single machine learning algorithms, or have limited sample sizes. Study aims mostly explore the impact of SO on prognosis or postoperative complications, resulting in monotonous research methods and conclusions. Pan-cancer and large sample studies are scarce. To overcome these limitations, our study utilized the public NHANES database to obtain a sufficiently large sample population with various cancer types, a long follow-up period, and adequate examination results.

Moreover, the factors associated with the onset and development of SO in cancer patients remain unclear. To fill the current research gap, our cross-sectional study constructed an effective and convenient machine-learning model to predict the presence of SO in cancer patients.

Moving to limitations, firstly, our study is cross-sectional, which provides lower proof strength than randomized controlled studies or cohort studies and limits the ability to infer causality. Future research should prioritize prospective cohort studies with systematic multi-omics data collection during long-term follow-up in order to better elucidate the causal mechanisms underlying SO among diverse populations. Secondly, this study extracted variables from the NHANES database, encompassing demographics, examination, laboratory, and questionnaire data. Multimodal data, including but not limited to non-contrast and contrast-enhanced CT and MRI, PET-CT or genomic features, or other multidimensional data, were not included as inputs in our machine learning models. Finally, the awareness of SO in clinical work is still not enough [38]. There is neither a consensus on the definition of SO nor widely recognized guidelines for cancer clinicians to follow in order to provide optimal treatment and nutritional care.

## 5. Conclusions

Our study highlights that SO is a risk factor for all-cause mortality in cancer patients. Our built LASSO model serves as a practical tool to identify SO patients who need early prevention and intervention to lower their mortality rate. In clinical practice, SO may emerge as a potential and promising target for cancer treatment strategies while laboratory research and clinical trials are further needed.

## Figures and Tables

**Table 1:** Difference in demographic and clinical characteristics of included participants with cancer.

	Overall (*n* = 1432)	Non-SO (*n* = 1221)	SO (*n* = 211)	*p*
Age (median (IQR))	62 (50–73)	60 (49–72)	71 (58–79.5)	<0.001
Sex = Male (%)	621 (43.4)	520 (42.6)	101 (47.9)	0.176
Race (%)				<0.001
Mexican American	112 (7.8)	69 (5.7)	43 (20.4)	
Non-Hispanic Black	187 (13.1)	180 (14.7)	7 (3.3)	
Non-Hispanic White	1044 (72.9)	895 (73.3)	149 (70.6)	
Other	89 (6.2)	77 (6.3)	12 (5.7)	
Education (%)				<0.001
Under high school	324 (22.6)	252 (20.7)	72 (34.1)	
High school or equivalent	345 (24.1)	294 (24.1)	51 (24.2)	
Above high school	762 (53.2)	674 (55.2)	88 (41.7)	
Marital status (%)				0.079
Widowed/divorced/separated	419 (29.8)	358 (29.8)	61 (29.6)	
Married/cohabiting	899 (63.8)	760 (63.2)	139 (67.5)	
Never married	90 (6.4)	84 (7.0)	6 (2.9)	
Poverty to income ratio (median (IQR))	2.4 (1.2–4.3)	2.9 (1.4–5)	2 (1.2–3.2)	<0.001
BMI (median (IQR))	27.4 (24.1–31.7)	26.9 (23.8–31.0)	30.6 (27.2–34.6)	<0.001
RFM (median (IQR))	35.7 (30.3–43.3)	35.2 (29.4–42.7)	41.9 (33.5–46.5)	<0.001
Smoking status (%)				<0.001
Former	563 (39.3)	454 (37.2)	109 (51.7)	
Never	599 (41.9)	514 (42.1)	85 (40.3)	
Now	269 (18.8)	252 (20.7)	17 (8.1)	
Alcohol consumption status (%)				0.198
Heavy	152 (18.5)	139 (19.0)	13 (14.0)	
Mild	505 (61.4)	440 (60.3)	65 (69.9)	
Moderate	166 (20.2)	151 (20.7)	15 (16.1)	
Glycemic control (%)				<0.001
DM	269 (18.8)	200 (16.4)	69 (32.7)	
IFG	66 (4.6)	56 (4.6)	10 (4.7)	
IGT	19 (1.3)	18 (1.5)	1 (0.5)	
Normal	1078 (75.3)	947 (77.6)	131 (62.1)	

Abbreviations: Non-SO, non-sarcopenic obesity; SO, sarcopenic obesity; BMI, body mass index; RFM, relative fat mass; DM, diabetes mellitus; IFG, impaired fasting glucose; IGT, impaired glucose tolerance. Poverty to income ratio (PIR) is a ratio of family income to poverty threshold: PIR > 1 indicates income above the poverty line; PIR = 1 denotes income at the poverty line; PIR < 1 reflects income below the poverty line. Continuous variables are displayed in the form of median (IQR).

**Table 2:** Difference in demographic and clinical characteristics according to the presence of sarcopenia and obesity.

	nS-nO (*n* = 574)	nS-O (*n* = 613)	S-O (*n* = 211)	S-nO (*n* = 34)	*p*
Age (median (IQR))	59 (48–72)	60 (49–70)	71 (58–79.5)	76.5 (71–81.75)	<0.001
Sex = Male (%)	301 (52.4)	193 (31.5)	101 (47.9)	26 (76.5)	<0.001
Race (%)					<0.001
Mexican American	27 (4.7)	40 (6.5)	43 (20.4)	2 (5.9)	
Non-Hispanic Black	73 (12.7)	106 (17.3)	7 (3.3)	1 (2.9)	
Non-Hispanic White	439 (76.5)	427 (69.7)	149 (70.6)	29 (85.3)	
Other	35 (6.1)	40 (6.5)	12 (5.7)	2 (5.9)	
Education (%)					<0.001
Under high school	101 (17.6)	139 (22.7)	72 (34.1)	12 (35.3)	
High school or equivalent	136 (23.7)	145 (23.7)	51 (24.2)	13 (38.2)	
Above high school	336 (58.6)	329 (53.7)	88 (41.7)	9 (26.5)	
Marital status (%)					0.008
Widowed/divorced/separated	142 (25.4)	205 (33.7)	61 (29.6)	11 (32.4)	
Married/cohabiting	371 (66.2)	367 (60.4)	139 (67.5)	22 (64.7)	
Never married	47 (8.4)	36 (5.9)	6 (2.9)	1 (2.9)	
Poverty to income ratio (median (IQR))	3.4 (1.6–5)	2.6 (1.3–4.3)	2 (1.2–3.2)	2 (1.3–3.3)	<0.001
BMI (median (IQR))	23.9 (21.7–25.7)	30.9 (28.2–34.9)	30.6 (27.2–34.6)	24.1 (23.5–25.3)	<0.001
RFM (median (IQR))	29.4 (26.8–35.7)	42.6 (34.2–45.7)	41.9 (33.5–46.5)	29.2 (27.3–30.0)	<0.001
Smoking status (%)					<0.001
Former	204 (35.6)	235 (38.3)	109 (51.7)	15 (44.1)	
Never	233 (40.7)	266 (43.4)	85 (40.3)	15 (44.1)	
Now	136 (23.7)	112 (18.3)	17 (8.1)	4 (11.8)	
Alcohol consumption status (%)					0.232
Heavy	65 (16.9)	72 (21.6)	13 (14.0)	2 (15.4)	
Mild	240 (62.5)	190 (57.1)	65 (69.9)	10 (76.9)	
Moderate	79 (20.6)	71 (21.3)	15 (16.1)	1 (7.7)	
Glycemic control (%)					<0.001
DM	62 (10.8)	134 (21.9)	69 (32.7)	4 (11.8)	
IFG	20 (3.5)	33 (5.4)	10 (4.7)	3 (8.8)	
IGT	5 (0.9)	13 (2.1)	1 (0.5)	0 (0.0)	
Normal	487 (84.8)	433 (70.6)	131 (62.1)	27 (79.4)	

Abbreviations: nS-nO, non-sarcopenia and non-obesity; nS-O, non-sarcopenia and obesity; S-O, sarcopenia and obesity; S-nO, sarcopenia and non-obesity; BMI, body mass index; RFM, relative fat mass; DM, diabetes mellitus; IFG, impaired fasting glucose; IGT, impaired glucose tolerance. Poverty to income ratio (PIR) is a ratio of family income to poverty threshold: PIR > 1 indicates income above the poverty line; PIR = 1 denotes income at the poverty line; and PIR < 1 reflects income below the poverty line. Continuous variables are displayed in the form of median (IQR).

**Table 3:** Hazard ratio of risk factors for all-cause mortality in univariable and multivariable Cox proportional hazard models in the entire cohort.

Model 1					Model 2				Model 3			
	HR	95%CIs Lower	95%CIs Upper	*p*	HR	95%CIs Lower	95%CIs Upper	*p*	HR	95%Cis Lower	95%CIs Upper	*p*
Dichotomous taxonomy of SO												
Non-SO	Reference				Reference				Reference			
SO	1.476	1.211	1.799	<0.001	1.367	1.122	1.666	0.002	1.368	1.107	1.690	0.004
Quadruple taxonomy of SO												
nS-nO	Reference				Reference				Reference			
nS-O	0.812	0.677	0.974	0.025	0.898	0.747	1.080	0.255	0.858	0.711	1.036	0.110
S-O	1.364	1.097	1.698	0.005	1.326	1.066	1.651	0.011	1.298	1.028	1.640	0.028
S-nO	2.002	1.335	3.004	<0.001	1.714	1.142	2.572	0.009	1.565	1.038	2.362	0.033

Abbreviation: HR, hazard ratio; 95CIs, corresponding 95% confidence intervals; Dichotomous taxonomy of SO: SO, sarcopenic obesity; Non-SO, non-sarcopenic obesity; Quadruple taxonomy of SO: nS-nO, non-sarcopenia and non-obesity; nS-O, non-sarcopenia and obesity; S-O, sarcopenia and obesity; S-nO, sarcopenia and non-obesity. Continuous variables are displayed as means (SD). Model 1 was unadjusted; Model 2 was adjusted for age and sex; Model 3 was adjusted for covariates in Model 2, plus race, education, and marital status.

**Table 4:** Hazard ratio of risk factors for all-cause mortality in univariable and multivariable Cox proportional hazard models among reproductive system groups (*n* = 459).

Model 1					Model 2				Model 3			
	HR	95%CIs Lower	95%CIs Upper	*p*	HR	95%CIs Lower	95%CIs Upper	*p*	HR	95%CIs Lower	95%CIs Upper	*p*
Dichotomous taxonomy of SO												
Non-SO	Reference				Reference				Reference			
SO	1.562	1.036	2.357	0.034	1.459	0.965	2.206	0.073	1.345	0.847	2.137	0.209
Quadruple taxonomy of SO												
nS-nO	Reference				Reference				Reference			
nS-O	1.265	0.845	1.893	0.254	1.200	0.801	1.797	0.376	1.117	0.742	1.682	0.595
S-O	1.836	1.124	2.999	0.015	1.661	1.014	2.721	0.044	1.464	0.855	2.506	0.321
S-nO	1.892	0.668	5.360	0.230	1.685	0.594	4.781	0.326	1.736	0.585	5.153	0.165

**Table 5:** Hazard ratio of risk factors for all-cause mortality in univariable and multivariable Cox proportional hazard models among integumentary system groups (*n* = 432).

Model 1					Model 2				Model 3			
	HR	95%CIs Lower	95%CIs Upper	*p*	HR	95%CIs Lower	95%CIs Upper	*p*	HR	95%Cis Lower	95%CIs Upper	*p*
Dichotomous taxonomy of SO												
Non-SO	Reference				Reference				Reference			
SO	1.612	1.102	2.359	0.014	1.520	1.038	2.226	0.031	1.318	0.882	1.970	0.178
Quadruple taxonomy of SO												
nS-nO	Reference				Reference				Reference			
nS-O	1.036	0.746	1.439	0.834	1.055	0.756	1.474	0.752	0.960	0.673	1.370	0.822
S-O	1.633	1.080	2.470	0.020	1.554	1.028	2.349	0.037	1.287	0.823	2.012	0.268
S-nO	0.665	0.092	4.799	0.686	0.684	0.095	4.947	0.707	0.861	0.118	6.283	0.883

**Table 6:** Hazard ratio of risk factors for all-cause mortality in univariable and multivariable Cox proportional hazard models among digestive system groups (*n* = 114).

Model 1					Model 2				Model 3			
	HR	95%CIs Lower	95%CIs Upper	*p*	HR	95%CIs Lower	95%CIs Upper	*p*	HR	95%CIs Lower	95%CIs Upper	*p*
Dichotomous taxonomy of SO												
Non-SO	Reference				Reference				Reference			
SO	1.083	0.587	1.999	0.797	1.081	0.586	1.993	0.804	1.554	0.776	3.112	0.213
Quadruple taxonomy of SO												
nS-nO	Reference				Reference				Reference			
nS-O	0.653	0.367	1.163	0.148	0.655	0.365	1.176	0.156	0.635	0.347	1.164	0.142
S-O	0.843	0.432	1.647	0.618	0.842	0.430	1.648	0.616	1.177	0.554	2.501	0.671
S-nO	0.502	0.151	1.663	0.259	0.502	0.151	1.663	0.259	0.455	0.131	1.582	0.216

**Table 7:** Hazard ratio of risk factors for all-cause mortality in univariable and multivariable Cox proportional hazard models among urinary system groups (*n* = 216).

Model 1					Model 2				Model 3			
	HR	95%CIs Lower	95%CIs Upper	*p*	HR	95%CIs Lower	95%CIs Upper	*p*	HR	95%CIs Lower	95%CIs Upper	*p*
Dichotomous taxonomy of SO												
Non-SO	Reference				Reference				Reference			
SO	1.024	0.687	1.527	0.908	1.117	0.747	1.668	0.590	0.968	0.614	1.526	0.889
Quadruple taxonomy of SO												
nS-nO	Reference				Reference				Reference			
nS-O	0.737	0.493	1.103	0.138	0.782	0.522	1.172	0.233	0.806	0.534	1.215	0.302
S-O	0.940	0.609	1.453	0.782	1.051	0.679	1.626	0.824	0.928	0.566	1.524	0.768
S-nO	1.473	0.808	2.687	0.207	1.430	0.783	2.609	0.245	1.577	0.855	2.908	0.144

**Table 8:** Hazard ratio of risk factors for all-cause mortality in univariable and multivariable Cox proportional hazard models among the other types of cancers (*n* = 73).

Model 1					Model 2				Model 3			
	HR	95%CIs Lower	95%CIs Upper	*p*	HR	95%CIs Lower	95%CIs Upper	*p*	HR	95%CIs Lower	95%CIs Upper	*p*
Dichotomous taxonomy of SO												
Non-SO	Reference				Reference				Reference			
SO	2.743	1.353	5.558	0.005	1.839	0.845	4.005	0.125	1.822	0.688	4.824	0.227
Quadruple taxonomy of SO												
nS-nO	Reference				Reference				Reference			
nS-O	1.598	0.683	3.742	0.280	1.330	0.543	3.259	0.533	0.762	0.257	2.258	0.623
S-O	3.521	1.480	8.377	0.004	2.098	0.853	5.160	0.107	1.565	0.503	4.871	0.439
S-nO	-	-	-	-	-	-	-	-	-	-	-	-

**Table 9:** Predictive performance of six machine learning models.

Model	Selected Features	Train	Test
XGBoost	All features	0.993	0.821
RF	All features	0.990	0.637
SVM	All features	0.742	0.633
	Top 10 features	0.622	0.605
	Top 15 features	0.615	0.587
	Top 20 features	0.624	0.587
Logistic Regression	All features	0.941	0.785
	Top 10 features	0.673	0.588
	Top 15 features	0.720	0.675
	Top 20 features	0.734	0.675
Stepwise Logistic Regression	All features	0.928	0.809
	Top 10 features	0.699	0.627
	Top 15 features	0.762	0.682
	Top 20 features	0.837	0.804
LASSO	All features	0.915	0.832
	Top 10 features	0.842	0.818
	Top 11 features	0.846	0.815
	Top 12 features	0.846	0.815
	Top 13 features	0.846	0.815
	Top 14 features	0.855	0.843
	Top 15 features	0.862	0.847
	Top 16 features	0.889	0.852
	Top 17 features	0.891	0.873
	Top 18 features	0.892	0.869
	Top 19 features	0.890	0.863
	Top 20 features	0.891	0.858

Abbreviations: RF, random forest; LASSO, least absolute shrinkage and selection operator; SVM, support vector machine; XGBoost, extreme gradient boosting.

**Table 10:** The descriptions of top 17 variables in optimal LASSO model.

Variable	Type	Coding and Explanation
Epilepsy (x_1_)	Categorical	1 = had history of epilepsy; 0 = no history of epilepsy
Anemia, Moderate (x_2_)	Categorical	1 = moderate anemia; 0 = mild anemia, non-anemia, or missing data
Race, Mexican American (x_3_)	Categorical	1 = Mexican American; 0 = Non-Hispanic Black, Non-Hispanic White, other
Parkinson (x_4_)	Categorical	1 = had history of Parkinson; 0 = no history of Parkinson
No history of heart attack (x_5_)	Categorical	1 = no history of heart attack; 0 = had history of heart attack or missing data
Race, Non-Hispanic Black (x_6_)	Categorical	1 = Non-Hispanic Black; 0 = Mexican American, Non-Hispanic White, other
Waist circumference (x_7_)	Continuous	Measured in centimeters (cm)
Stroke (x_8_)	Categorical	1 = no stroke history; 0 = had stroke or missing data
Upper arm length (x_9_)	Continuous	Measured in centimeters (cm)
MAO (x_10_)	Categorical	1 = diagnosed as metabolically abnormal obese; 0 = no metabolically abnormal obese or missing data
Congestive heart failure (x_11_)	Categorical	1 = had congestive heart failure; 0 = no congestive heart failure or missing data
Age (x_12_)	Continuous	Measured in years, integer
Upper leg length (x_13_)	Continuous	Measured in centimeters (cm)
Smoke now (x_14_)	Categorical	1 = current smoker; 0 = former smoker, never smoked, or missing data
Military (x_15_)	Categorical	1 = participant has prior service in the armed forces; 0 = participant has no prior service in the armed forces
ABSI (x_16_)	Continuous	ABSI = (waist circumference / hip circumference) ÷ BMI
Heart attack (x_17_)	Categorical	1 = had history of heart attack; 0 = no history of heart attack or missing data

MAO: metabolically abnormal obese. Individuals can be diagnosed as MAO when they meet three or more of the following criteria: waist circumference (WC) of ≥102 cm for men and ≥88 cm for women; triglyceride levels of ≥1.69 mmol/L; HDL cholesterol levels of <1.04 mmol/L for men and <1.29 mmol/L for women; blood pressure with systolic BP ≥130 mmHg or diastolic BP ≥85 mmHg; and fasting plasma glucose of ≥5.6 mmol/L [19]. ABSI: a body shape index. ABSI is an anthropometric marker of central obesity, reflecting the degree of abdominal fat deposition. It has already been established that ABSI may indicate decreased muscle mass and shows potential as an early diagnostic tool for sarcopenic obesity [20].

## Data Availability

Data available in a publicly accessible repository:The data presented in this study are openly available in the National Health and Nutrition Examination Survey at https://www.cdc.gov/nchs/nhanes/.
